# Strigolactones and abscisic acid interactions affect plant development and response to abiotic stresses

**DOI:** 10.1186/s12870-023-04332-6

**Published:** 2023-06-13

**Authors:** Magdalena Korek, Marek Marzec

**Affiliations:** Institute of Biology, Biotechnology and Environmental Protection, Faculty of Natural Sciences, University of Silesia in Katowice, Jagiellonska 28, Katowice, 40-032 Poland

**Keywords:** Abiotic stress, Abscisic acid, Phytohormone cross-talk, Plant development, Strigolactones

## Abstract

Strigolactones (SL) are the youngest group of plant hormones responsible for shaping plant architecture, especially the branching of shoots. However, recent studies provided new insights into the functioning of SL, confirming their participation in regulating the plant response to various types of abiotic stresses, including water deficit, soil salinity and osmotic stress. On the other hand, abscisic acid (ABA), commonly referred as a stress hormone, is the molecule that crucially controls the plant response to adverse environmental conditions. Since the SL and ABA share a common precursor in their biosynthetic pathways, the interaction between both phytohormones has been largely studied in the literature. Under optimal growth conditions, the balance between ABA and SL content is maintained to ensure proper plant development. At the same time, the water deficit tends to inhibit SL accumulation in the roots, which serves as a sensing mechanism for drought, and empowers the ABA production, which is necessary for plant defense responses. The SL-ABA cross-talk at the signaling level, especially regarding the closing of the stomata under drought conditions, still remains poorly understood. Enhanced SL content in shoots is likely to stimulate the plant sensitivity to ABA, thus reducing the stomatal conductance and improving the plant survival rate. Besides, it was proposed that SL might promote the closing of stomata in an ABA-independent way. Here, we summarize the current knowledge regarding the SL and ABA interactions by providing new insights into the function, perception and regulation of both phytohormones during abiotic stress response of plants, as well as revealing the gaps in the current knowledge of SL-ABA cross-talk.

**Supplementary Information:**

The online version contains supplementary material available at 10.1186/s12870-023-04332-6.

## Background

Phytohormones (plant hormones) are a group of naturally occurring, organic chemical compounds produced by plants in micromolar concentrations however, they significantly affect the entire life cycle of plants, from early embryogenesis to senescence [1]. Plant hormones act as chemical messengers coordinating the molecular pathways that lead to the growth and development of the organisms. Several members of the phytohormone family have already been identified, including abscisic acid (ABA), auxins (AUX), brassinosteroids (BR), cytokinins (CKs), ethylene (ET), gibberellins (GA), jasmonates (JA), and strigolactones (SL) [2]. Due to the sessile lifestyle, plants are constantly subjected to a wide range of biotic and abiotic stresses [3]. To adapt to such adverse situations, plants developed various mechanisms that allow them to perceive the stress stimulus and consequently to provide adequate defense reactions. When faced with unfavourable environmental conditions, plants require the activation of a complex signaling network, where phytohormones play a critical role [4]. Interestingly, individual hormones can interact with each other to ensure plant stress tolerance. These interactions can occur at the hormone biosynthesis or signaling level and could be both stimulatory and inhibitory in nature [5–7]. Here, we present a comprehensive overview of the cross-talk between ABA, commonly referred to as the stress hormone, and SL, the youngest member of phytohormone family, which is increasingly confirmed to play a role in the plant’s response to abiotic stresses.

## SL: a brief overview

Initially identified as rhizosphere signaling molecules, SL were first identified from cotton (*Gossypium arboreum*) root exudate in the 1960s and were found to induce germination of parasitic seeds such as the witchweeds (*Striga spp*.) and broomrapes (*Orobanche* and *Phelipanche spp*) [8]. For this reason, the recognized molecule was named strigol. Later, it was shown that SL exuded by plant roots trigger hyphae branching of mycorrhizal fungi, thus increasing the chances of contact between symbionts [9]. More recent studies provided a better understanding of SL function as a direct regulator of plant growth. In 2008, the inclusion of SL in the list of plant hormones was supported by the analysis of mutants that exhibited semi-dwarf and highly shoot branching phenotypes in three genetically distant model plant species, such as arabidopsis (*Arabidopsis thaliana*), pea (*Pisum sativum*), and rice (*Oryza sativa*) [10, 11]. The studies confirmed that treatment with a synthetic analogous of SL rescued the phenotype of SL-depleted plants, which was not possible with SL-insensitive mutants. Further, the impact of SL on shaping the above-ground plant architecture was also proved in other species [12, 13]. Up to now, semi-dwarf and highly branched mutants affected in SL-biosynthesis or signaling pathway have been identified from a wide range of species, including arabidopsis (*more axillary growth, max*) [14–17], petunia (*Petunia hybrid*; d*ecreased apical dominance, dad*) [18–22], pea (*Pisum sativum; ramousus, rms*) [23, 24] and rice (*high-tillering dwarf, htd; dwarf, d*) [25, 26].

**Fig. 1:**
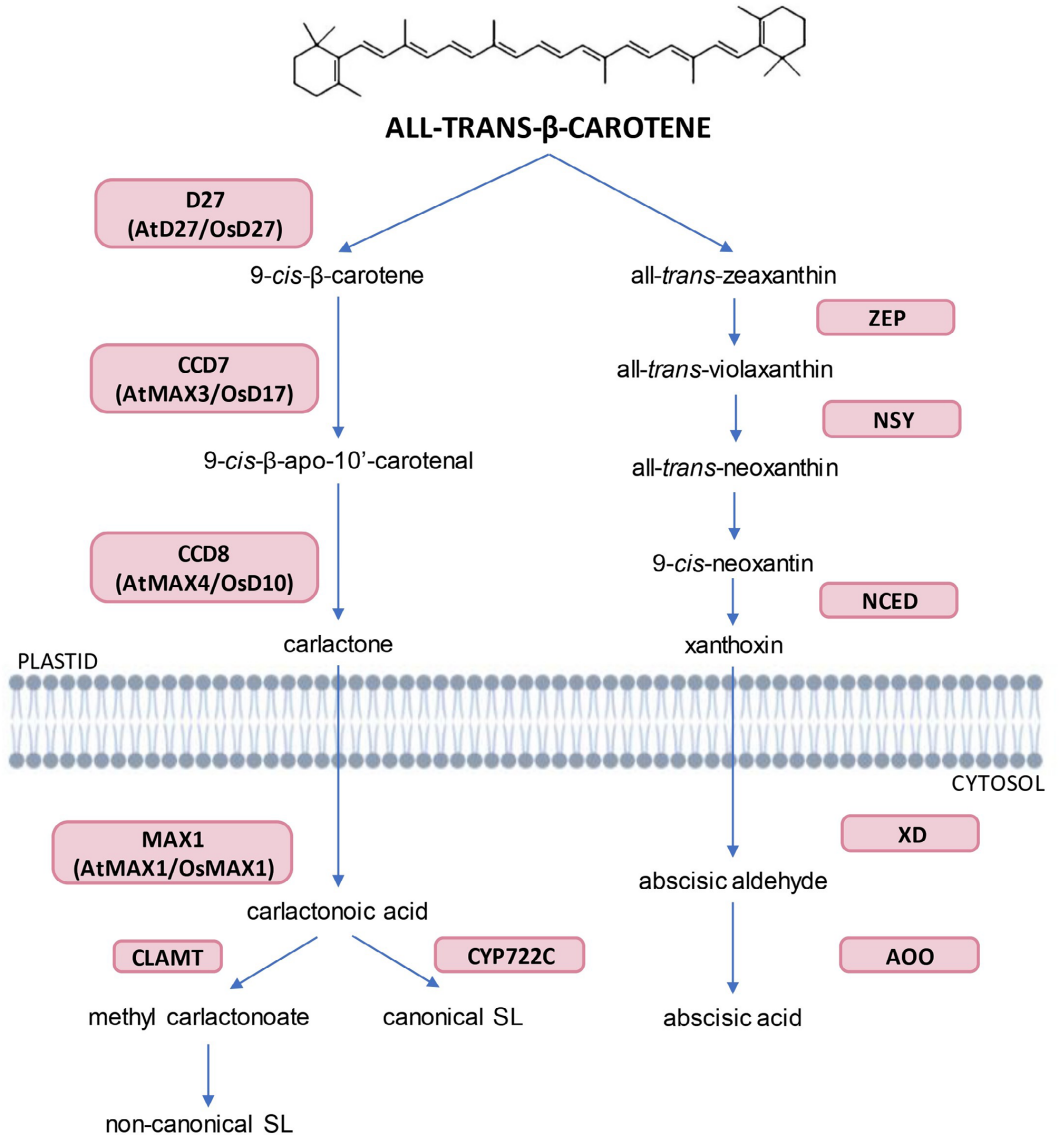
The biosynthetic pathways of strigolactones (SL) and abscisic acid (ABA) share a common precursor. The formation of SL starts with the isomerization of all-*trans*-β-carotene by the DWARF 27 (D27) at the C-9 position. Next, two CAROTENOID CLEAVAGE DIOXYGENASEs – CCD7 and CDD8 convert 9-*cis*-β-carotene to carlactone, which is further oxidized by cytochrome P450 monooxygenases, such as MORE AXILLARY GROWTH 1 (MAX1). The carlactonoic acid (CLA) undergoes further reactions either by CLA methyltransferase (CLAMT) to form a methyl carlactonoate, which is a key intermediate for non-canonical SL, or by enzymes from CYP722C subfamily producing canonical SL. The ABA biosynthesis part that takes place in the plastid requires a series of enzymatic reactions that lead to the formation of xanthoxin. Then xanthoxin is transported to cytosol, converted to abscisic aldehyde by XANTHOXIN DEHYDROGENASE (XD), and further oxidized by ABSCISIC ALDEHYDE OXIDASE (AAO) to ABA. Created with BioRender.com

SL are primarily synthesized in the roots and subsequently transported to the above-ground parts of the plant [27]. The initial step in SL biosynthesis is the conversion of all-*trans*-β-carotene to carlactone (Fig. 1). This process is carried out in plastids and involves three enzyme players - carotenoid isomerase (D27) and two carotenoid cleavage dioxygenases (CAROTENOID CLEAVAGE DIOXYGENASE7/8; CCD7, CCD8) [28]. Another step occurs in the cytoplasm and is led by MAX1-type monooxygenase, transforming carlactone into carlactonoic acid (CLA), giving rise to other SL and SL-like compounds. The subsequent steps of SL biosynthesis vary across plant species [29]. In arabidopsis, maize (*Zea mays*) and tomato (*Solanum lycopersicum*) research, it was revealed that carlactonoic acid is further transformed by CLA methyltransferase (CLAMT) to methyl carlactonoate (MeCLA), which is the key intermediate for non-canonical SL [30]. On the other hand, enzymes from the CYP722C subfamily have been shown to form canonical SL in cowpea (*Vigna unguiculate*), tomato, cotton, and Lotus japonicus [31]. Canonical SL have a tricyclic lactone structure composed of three rings (ABC-rings) connected to a butenolide group (D-ring) via an enol-ether bridge [32]. Rings A and B differ due to the additional functional groups (i.e. −CH_3_, −OH, −C(O)CH_3_), while rings C and D are highly conserved and play an essential role in the biological activity of SL molecules [33]. Canonical SL are further divided into strigol- and orobanchol-type classes based on the stereochemistry of C-ring, which may be a β- and an α-oriented, respectively [34]. At the same time, both subgroups share the 2’R orientation [35]. In the research area, the most commonly used synthetic analogue of SL is *rac*-GR24. This compound is an equimolar mixture of the two enantiomers: GR24^5DS^ that mimics the configuration and activity of the natural 5-deoxystrigol (5DS) and GR24^*ent* − 5DS^ with stereochemistry at 2’*S* not occurring in natural SL [27]. During the chemical synthesis of GR24, the two orobanchol-type enantiomers are also produced however, these compounds are not usually involved in biological assay [36]. It is crucial that GR24^*ent* − 5DS^ is also perceived by KARRIKIN INSENSITIVE 2 (KAI2), a receptor involved in karrikin (KAR) signaling. Thus the results obtained with the usage of *rac*-GR24 might be ambiguous due to the stimulation of both SL and KAR pathways [36]. To activate the SL transduction exclusively, the use GR24^5DS^ or recently synthetized GR24^4DO^ is recomended [37]. In contrast to canonical SL, non-canonical SL are very diverse in the structure of their ABC-rings, but possess both an enol-ether bridge and D-ring moieties. Studies have demonstrated that a single plant species can generate various types of SL [38]. Furthermore, it has been suggested that SL can result in different physiological responses in plants depending on their chemical composition [39–41]. The fact that canonical SL are found only in limited plant species, and their specific and stereoselective movement from roots to shoots, indicates that the plant hormones responsible for suppressing shoot branching might be non-canonical SL, and not canonical SL [41–43]. To date, more than 30 naturally occurring SL have been identified among mono- and dicotyledonous plants serving many roles in plant growth and development [29]. Experimental studies have confirmed the involvement of SL in a range of processes such as parasitic seed germination, early seedling development, leaf senescence and control of main and lateral root or root-hair elongation [44, 45]. Besides these developmental processes, there is a growing body of evidence suggesting that SL also participate in the plant’s response to various biotic and abiotic stresses. Specifically, the activity of SL has been documented during the plant’s response to suboptimal environmental conditions such as drought, salinity, high or low temperature, nutrient deficiency, oxidative stress, and fluctuations in light quality and intensity [46, 47]. Moreover, there have been postulations about the potential role of SL in plant’s defense to pathogens [48]. Recent reports have shed light on the molecular mechanisms underlying the involvement of SL in stress responses, highlighting their potential as targets for improving plant tolerance to environmental stressors [31, 49].

**Fig. 2:**
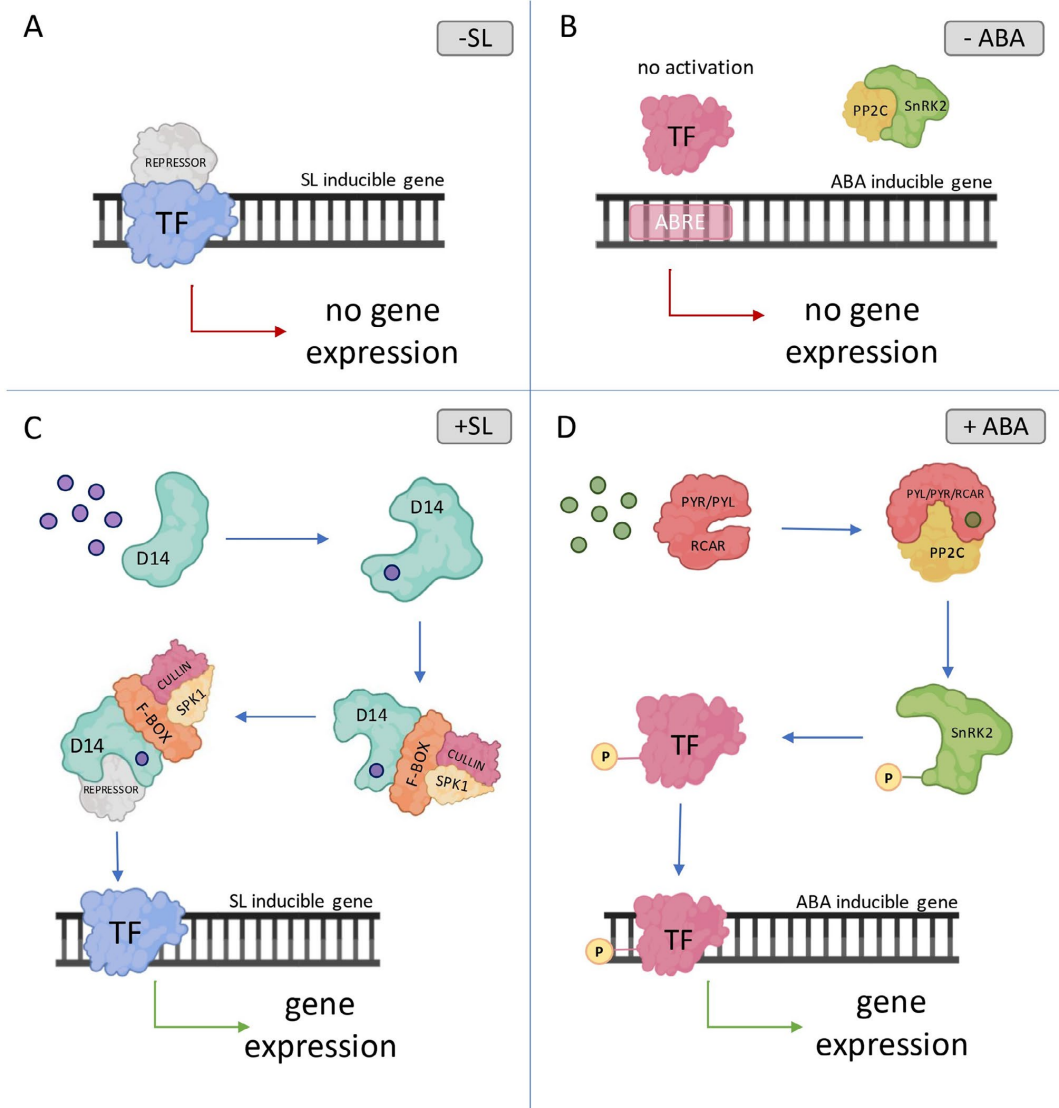
Perception and signaling of strigolactones (SL) and abscisic acid (ABA). **A**) In the absence of SL, the expression of SL inducible gene is blocked by repressor. **C**) The SL molecules are recognized and bound by D14 protein, which results in conformational changes of SL receptor. Following, the D14 protein interacts with the F-box protein from the SCF complex and the SL repressor, resulting in degradation of SL repressor. As a consequence, the transcription of SL inducible gene is activated. **B**) In the absence of ABA, the TF remains inactive as the interaction between PP2C and SnRK2 blocks its phosphorylation. **D**) When ABA molecules are recognized and bound by ABA receptor (PYL/PYR/RCAR), the receptor undergoes a conformational change. This change enables the ABA receptor to interact with the PP2C protein, which then releases the SnRK2. The SnRK2 is subsequently autophosphorylated or phosphorylated by other proteins, resulting in the activation of TF. Once activated, the TF can bind to ABRE elements in the promoter of ABA inducible gene and recruit transcriptional machinery. TF – transcription factor, PP2C - PROTEIN PHOSPHATASE 2 C, PYR - PYRABACITN RESISTANCE, PYL - PYRABACTIN RESISTANCE 1-LIKE, RCAR - REGULATORY COMPONENT OF ABA RECEPTOR, SnRK2 - SUCROSE NONFERMENTING 1 RELATED PROTEIN KINASES 2, ABRE – ABA responsive element, D14 – DWARF 14, SCF – SPK1-CULLIN-F-BOX, P – phosphorus residue. Created with BioRender.com

In the last decade, various breakthroughs have been made in scientific research regarding the perception and signaling of the SL. All major SL signal transduction pathways components were already described in arabidopsis and rice [50]. Similar to most phytohormones, the mechanism for transducing the SL signal is based on the degradation of repressor protein (Fig. 2A). The first step of the cascade perception is recognizing and binding the SL molecules by the receptor (AtD14/OsD14), which belongs to the 𝛼/𝛽 hydrolase protein family [51] (Fig. 2B). This interaction results in conformation changes of the D14, which is necessary for the interaction between receptor and other components from SL signaling complex [52]. The receptor with altered conformation can bind the F-box protein (AtMAX2/OsD3) from the SKP1-CULLIN-F-BOX complex (SCF) and the SL repressor (SUPPRESSORS OF MAX2 1-LIKE6, 7, 8, AtSMXL6,7,8/OsD53) [53]. Following, the degradation of the SL repressor results in the activation of transcription factors (TFs) related to SL [54]. Recently, *Arabidopsis* transcriptomic studies revealed that exogenous SL may activate 24 genes and repress 14 genes encoding TFs, respectively. The effect of SL-dependent responsiveness was experimentally confirmed in three of them – *BRANCHED1* (*BRC1*), *TCP DOMAIN PROTEIN1* (*TCP1*) and *PRODUCTION OF ANTHOCYANIN PIGMENT1* (*PAP1*), whose roles are related to the control of shoot branching, leaf shape, and anthocyanin biosynthesis [55]. Interestingly, it was also shown that SMXL6 targeted promoter regions of *SMXL6,7,8*, indicating that this SL repressor protein functions as a self-regulating TF, which may also control the expression of other *SMXL* genes.

## ABA: a brief overview

Abscisic acid (ABA) was discovered in the early 1960s by two independent research groups from the United States and the United Kingdom. While Eagles et al. identified a molecule that can trigger dormancy and called it dormin [56], Ohkuma et al. isolated an abscission-accelerating factor from cotton fruits, which they called abscisin II [57]. Both discovered chemical compounds turned out to have the same chemical structure [58]. Therefore, the newly-recognized molecule was renamed abscisic acid to standardise the nomenclature. In contrast to SL, the structure of ABA is conserved through plant kingdom [35]. From a chemical point of view, ABA is a 15-carbon molecule classified as a sesquiterpenoid formed by joining three isoprenoid units [59]. The *trans*- or *cis*- stereoisomerization is determined by the orientation of the carboxyl moiety at position 2’. Moreover, the presence of an asymmetric carbon atom 1’ decides about the S(+) or R(-) enantiomers [60]. Naturally occurring ABA is mainly found in plants as (S)-*cis*-ABA [61]. ABA is mostly synthesized in mature leaves (phloem companion cells, guard cells, and mesophyll cells), but also in roots, flowers, fruits, and seeds [62]. Due to specific phenotype such as precocious germination of seeds and wilted appearance of the plants, mutants insufficient in ABA biosynthesis were isolated from numerous plant species, including arabidopsis, barley (*Hordeum vulgare*), tomato, tobacco (*Nicotiana tabacum*) and maize [63]. ABA, similarly to SL, is a derivative of all-*trans*-β-carotene, thus the first steps of enzymatic reactions take place in plastids (Fig. 1). The process starts with the hydroxylation of all-*trans*-β-carotene to all-*trans*-zeaxanthin, which is later converted to all-*trans*-violaxanthin by ZEAXANTIN EPOXIDASE (ZEP) [64]. Following, NEOXANTIN SYNTHETASE (NSY) transforms all-*trans*-violaxanthin to all-*trans*-neoxanthin, then isomerized to 9-*cis*-neoxantin [65]. The last step of the biosynthetic pathway that occurs in the plastids is led by EPOXYCAROTENOID DIOXYGENASE (NCED) and results in cleavage of 9-*cis*-neoxanthin to xanthoxin (Fig. 1). This is the only non-reversible reaction and is believed to be a key rate-limiting point in the biosynthesis process [66]. Further, xanthoxin is transported to the cytosol, where it is converted to abscisic aldehyde by XANTHOXIN DEHYDROGENASE (XD). The final step is led by ABSCISIC ALDEHYDE OXIDASE (AAO) and results in oxidation of abscisic aldehyde to ABA (Fig. 1) [67].

It has become progressively clear that ABA plays a dual role in the plants’ life cycle as a plant growth regulator and an improving stress tolerance factor depending on the relative endogenous concentrations of ABA [62]. Under optimal environmental conditions, it has been demonstrated that low concentrations of ABA regulate plants’ vegetative growth, including seed development and germination, embryo maturation, root architecture, bud dormancy, fruit ripening, and leaf abscission [68]. Conversely, enhanced amounts of ABA play an essential role in plants’ adaptation to a varied range of stresses such as heat or cold stress, high level of solid salinity, and abundant heavy metals [69]. One of the most well-known and fundamental actions of ABA is to control the stomatal closure during drought stress, which is critical for maintaining water retention in the plant [70]. As the main phytohormone acting against abiotic stresses, the fluctuation of endogenous ABA levels must be consistently triggered by the balance between biosynthesis and catabolism due to changing environmental conditions [71]. ABA catabolism is generally categorized into two types of reactions, conjugation and hydroxylation [72]. The most widespread form of conjugated ABA is ABA-glucosyl ester (ABA-GE), which is biologically inactive. However, recent studies indicate that ABA-GE may act as a reservoir of active ABA in dehydration conditions through one-step hydrolysis by β-glucosidase [73]. The predominant and non-reversible enzymatic reaction leading to ABA catabolism is 8’-hydroxylation led by CYP707As, cytochrome P450 monooxygenases.

The pathway for ABA signal transduction requires three main classes of proteins; ABA receptors named PYRABACITN RESISTANCE/PYRABACTIN RESISTANCE 1-LIKE/REGULATORY COMPONENT OF ABA RECEPTOR (PYR/PYL/RCAR), ABA repressors from the PROTEIN PHOSPHATASE 2C (PP2Cs) group A family, and the SUCROSE NONFERMENTING 1 RELATED PROTEIN KINASES 2 (SnRK2s) as a positive regulators [74]. When ABA is absent, a physical association exists between PP2Cs and SnRK2s. This interaction has an inhibitory effect on the phosphorylation activity of SnRK2s. Consequently, ABA signal transduction is blocked, preventing the activation of downstream TFs [59] (Fig. 2C). In the case of ABA presence, the hormone is perceived and bound by PYR/PYL/RCAR receptors, which changes the receptor’s conformation and allows for the interaction between receptor and PP2Cs catalytic site. This interaction suppresses the phosphatase activity of ABA repressor proteins and relieves the inhibition of SnRK2s [75]. The released SnRK2s are then activated by autophosphorylation or phosphorylation by other proteins, and further SnRK2s are able to phosphorylate downstream proteins or TFs that induce ABA responses [76] (Fig. 2D). The activated ABA-related TFs directly bind to ABA-responsive element (ABRE) – (ACGTGG/TC), a major *cis*-element in the promoters of ABA-responsive genes [77]. The phosphorylation/dephosphorylation is a key process controlling ABA signal transduction and activation of ABA-responsive genes. In addition, ubiquitination and degradation of core proteins in ABA signaling pathway by the ubiquitin proteasome system (UPS) is also a critical step that modulates the signal relay [78]. Protein degradation by the UPS is a regulatory mechanism studied during various aspects of ABA stress response. So far, over 20 proteins with E3 ligase activity have been identified that regulate the protein level of ABA signaling core components, including ABA receptors, PP2Cs proteins and ABA-responsive TFs [79].

## Interactions between SL and ABA biosynthesis pathways during plant growth and development

All-*trans*-β-carotene is a molecule that undergoes a cascade of enzymatic reactions leading to the formation of both SL and ABA phytohormones (Fig. 1). The *TILLERING 20* (*T20*) gene, which encodes an isomerase involved in carotenoid biosynthesis has been functionally analyzed to prove that SL and ABA share a common precursor. Loss-of-function mutation in the *T20* gene reduced both SL and ABA levels in rice plants [80]. Therefore, it raises the question of whether SL and ABA interact with each other at the biosynthetic level to maintain hormone homeostasis.

In 2015 an *in silico* analysis showed that *cis*-regulatory elements in promoters of arabidopsis and rice SL biosynthesis genes are related to hormonal regulation [81]. Most of them are connected with ABA-responsive factors, which clearly emphasizes that the biosynthesis of SL may be ABA-dependent. Indeed, several reports on various plant species suggest the role of ABA in regulating SL biosynthesis. The spatial-temporal expression pattern of a reporter gene controlled by the native AtD27 promoter (*pAtD27:NLS-GUS*) enhanced in primary and lateral roots of 7-day-old arabidopsis seedlings after ABA treatment. RT-qPCR further confirmed this observation, showing an increase in *AtD27* expression caused by ABA application [82] (Supplementary Table 1). In another research, a noteworthy increase in the relative transcripts levels of arabidopsis *CCD7* and *CCD8* SL-biosynthesis genes in leaves was observed 1 h after ABA treatment, with the maximum level of increased expression of both genes reached after 10 hours [83]. Similar correlations were observed for tomato seedlings, where treatment with NCED inhibitor abamineSG reduced ABA and SL content in roots compared to non-treated plants [84]. Comparable results were also found in tomato ABA-deficient mutants, such as *notabilis* (mutation in *NCED* gene), *sitiens* and *flacca* (mutations in *AAO* enzyme). The endogenous content of both SL and ABA was much lower in analyzed mutants than in wild-type (WT) plants [84]. In contrast, applying the carotenoid cleavage dioxygenase inhibitor D2 reduced SL but not ABA content in roots [84]. The effect of limiting SL biosynthesis due to inhibited ABA production was also noted in monocotyledonous plants. The root exudates of maize plants with a null mutation in the *ZmNCED1* gene contributed to a significant reduction in the germination of parasitic seeds, and this outcome is suggested to be a result of low SL content [85]. All this together clearly highlights the positive impact of ABA on SL biosynthesis under optimal plant growth conditions. Notably, a stimulating effect of SL on the activity of ABA biosynthesis genes was also demonstrated. In rice, five NCED genes are believed to be involved in ABA biosynthesis [86]. After treating rice seedlings with *rac*-GR24, the expression of *OsNCED1* and *OsNCED2* was significantly induced in shoot bases, while the activity of *OsNCED3* was enhanced in roots. However, the expression level of *OsNCED4 and OsNCED5* remained unchanged [80] (Supplementary Table 1). These results suggest that different *NCED* genes might be involved in ABA biosynthesis in an organ-specific manner, and some may be SL-activated.

Since SL and ABA share a common precursor, it was initially assumed that their relationship should be competitive rather than promoting. However, recent research showed that D27 might also stimulate ABA biosynthesis. The shoot ABA content was significantly increased in two independent rice lines overexpressing the *OsD27* gene compared with WT. Furthermore, it was observed that mutation in the *OsD27* gene resulted in untouched ABA levels in rice shoots, in contrast to other SL-deficient mutants, where ABA accumulation was increased [87]. Interestingly, the induced expression of the *OsD27* gene was demonstrated in both *Osccd7* and *Osccd8* mutants. If *D27* actually promotes ABA amounts, then the enhanced levels of *D27* transcripts followed by increased levels of ABA in *osccd7/8* mutants could be explained with positive feedback of SL deficiency on Os*D27* expression. The authors could not explain the mechanism by which D27 controls ABA levels in rice. The in vitro experiment ruled out the possibility that D27 is directly involved in forming intermediates in ABA biosynthetic pathway (9’-*cis* violaxanthin or 9’-*cis*-neoxanthin) from their all-*trans* precursors [88]. In arabidopsis, AtD27 has two closely related homologs, D27-LIKE1 and D27-LIKE2, which might also be involved in β-carotene isomerization [89, 90]. Plants with a mutation in *D27-LIKE1* gene do not present phenotypes typical for SL-depleted or SL-insensitive mutants. However, the overexpression line (*OE-D27LIKE1*) in the background of the *d27* mutant restored the more-branching phenotype, indicating the participation of AtD27-LIKE1 in SL biosynthesis [90]. More importantly, the in vitro assay showed that D27-LIKE1 displayed an affinity for all-β-carotene isoforms and accepted zeaxanthin and violaxanthin as substrates, showing that D27-LIKE1 might affect both ABA and SL content [89]. It was proposed that D27/D27-LIKE1 might indirectly control the relationship between SL and ABA biosynthetic pathways. In line with this suggestion is a study showing increased ABA concentrations in 6-week-old leaves of transgenic barley with *HvD27* gene under arabidopsis promoter AtD27 (*pAtD27::HvD27*) [91]. Moreover, the *atd27* mutant showed about 20% less ABA in shoots than WT [82]. Noteworthy, the researchers did not detect a significant difference in root samples both in rice and arabidopsis. The analysis of the overexpression of other genes involved in SL biosynthesis was also investigated regarding ABA accumulation. The increased shoot ABA levels were observed in arabidopsis transgenic lines overexpressing the soybean (*Glycine max*) orthologs of *AtCCD7*, *AtCCD8* and *AtMAX1* genes [92] (Supplementary Table 1). Thus, enhanced production of SL seems to promote ABA content in the shoot. On the other hand, the same research revealed that mutation in one of the arabidopsis *AtCCD7*, *AtCCD8* or, *AtMAX1* genes results in decreased ABA content. This observation is in contrast to rice studies [87] therefore, the role of particular genes involved in SL biosynthesis pathway remains elusive and requires further in-deep investigations both in monocots and dicots species.

Despite numerous studies indicating the mutual promotion of SL and ABA biosynthesis, scientists also indicated a possible antagonistic effect on the production of both phytohormones. In mature barley roots, elevated ABA levels by RNAi-mediated down-regulation of two ABA catabolic genes coding ABA 8’-hydroxlase (*HvABA8’OH-1* and *HvABA8’OH-3*) resulted in lower amounts of *HvD27*, *HvCCD7*, *HvCCD8*, and *HvMAX1* transcripts in two independent transgenic lines (LOHi236 and LOHi272). The limited synthesis of SL contributed to the high-tillering phenotype of RNAi mutants, suggesting that in WT plants, the homeostasis between ABA and SL is essential for controlling the tiller formation [91]. The negative impact of elevated ABA concentration on SL biosynthesis genes expression was also proved in 2-week-old rice seedlings. Application of ABA strongly repressed expression of *OsCCD8* and *OsD27* genes in roots 3, 6, and 12 h after treatment and moderately reduced *OsCCD7* expression after 12 h. Consistent with the inhibition of SL biosynthetic by ABA, expression of SL repressor *OsD53* was also significantly reduced 6 and 12 h after ABA teratment [80]. On the other hand, the negative impact of SL treatment on ABA content was also detected. In the germination assay of *Pelipanche ramosa* parasitic seeds, it is hypothesized that GR24 stimulate the ABA degradation by strongly up-regulating the *PrABA8’OH-1* gene, thereby promoting seed germination [93]. Another study corroborated this discovery, showing that the application of GR24 decreases the promoter DNA methylations of this ABA catabolic gene, promoting its expression [94]. Thus, it may be assumed that SL found in root exudates of hosting plants are a germination signal for parasitic seeds and promote their germination by degradation of ABA. Finally, the application of *rac*-GR24 markedly inhibited the ABA-induced accumulation of sugars and anthocyanins in *Vitis vinifera* (grape) berries attached to plants [95]. To summarize, the data collected indicate that changes in SL and ABA levels in plants are influenced by several factors, including the organ type and the stage of the plant’s life cycle, under ideal growth conditions. The interaction between SL and ABA can either promote or hinder the production of each other, resulting in a balance of both phytohormones and triggering an unprecedented plant response.

## Interplay in SL and ABA biosynthesis pathways under abiotic stresses

Abiotic stresses such as drought, salinity, extremes of temperatures, or nutrient starvation pose a severe threat to plant growth and development, reflected in worldwide crop losses and threatening food security [96, 97]. Therefore, designing new strategies to enhance plants’ adaptation to harsh circumstances is crucial. One promising approach is to comprehensively understand the phytohormone biosynthetic pathways, which play a key role in regulating plant responses to environmental stresses [98, 99]. Undoubtedly, the most well-known hormone involved in plant responses to various abiotic stresses is ABA, referred to in the literature as the stress hormone [100]. ABA rapidly accumulates to high levels during unfavourable environmental conditions, such as water deficit, soil salinity and osmotic stress, which alters the expression profile of TFs and related stress-responsive genes [101]. On the other hand, more and more research studies have evidenced a clear-cut role of SL in conferring abiotic stress tolerance across plant species.

**Fig. 3:**
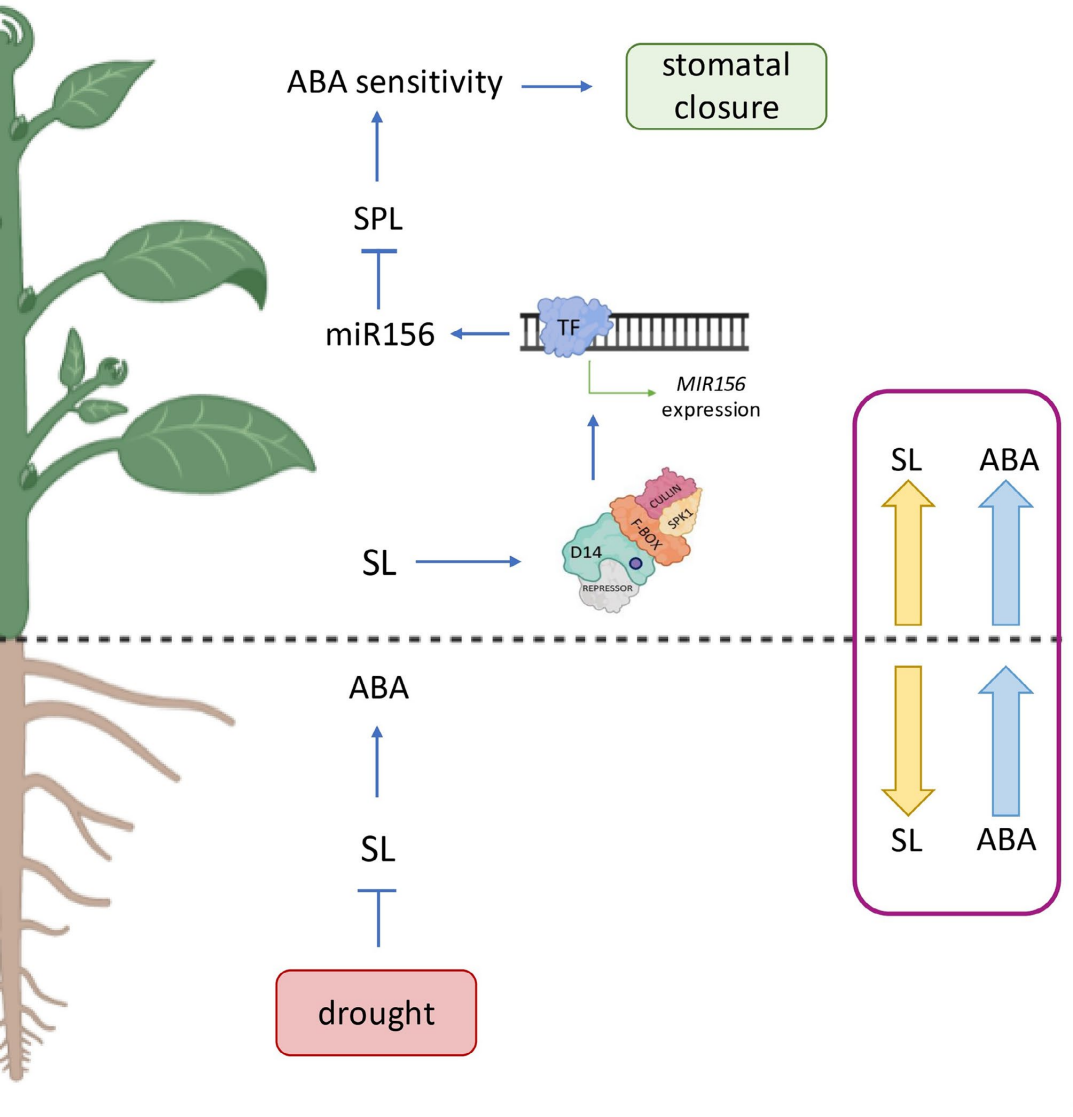
The model of ABA and SL organ-specific relations under drought. In the below-ground organ part of plants the relations between SL and ABA are concentrated on the biosynthesis level. The drop of SL content in roots plays a sensor role of plant stress and promote ABA accumulation, thus activating the plant resistance mechanisms. In the shoots, enhanced SL biosynthesis leads to the degradation of SL repressor through the assembly of the D14-SCF complex. This, in turn, activates the expression of the *MIR156* gene, resulting in the accumulation of mature miR156 molecules that inhibit mRNA translation. This process ultimately prevents the formation of SPL native proteins, making guard cells more sensitive to ABA and accelerating their closure. The blue and yellow arrows indicate the content of ABA or SL in each plant organ during drought stress. D14 – DWARF 14, SCF – SKP1-CULLIN-F-BOX, TF – transcription factor, SPL - SQUAMOSA PROMOTER BINDING PROTEIN-LIKE. Created with BioRender.com

It was shown that SL application improves the resistance of WT plants to drought stress in arabidopsis [61], wheat [77], maize [78], lettuce (*Lactuca sativa*), and tomato [79]. What is more, 3-week-old rice seedlings harbouring the mutation in the *T20* gene, which results in both lower SL and ABA concentrations, were much more sensitive to various types of stresses (osmotic stress, salt stress, dehydration, and cold tolerance) than WT plants [80]. Considering all these facts, researchers are targeting SL and ABA cooperation in abiotic stress resistance plants’ mechanisms. Using the parameter of 50% inhibition of seed germination by thermo-inhibition (TI_50_) it was shown that arabidopsis *max1* and *max2* mutants are 3 °C more sensitive to temperature than WT seeds. The application of *rac*-GR24 increased the TI_50_ of WT, *ccd7* and, *max1*, but not *max2*, revealing that hypersensitivity to heat stress is SL-dependent [102]. The effect of rescuing the phenotype of high temperature-sensitive seeds by *rac*-GR24 application was possible due to decreasing the ABA\GA ratio via suppression of heat-induced ABA increase. The lower ABA content triggered by SL was due to the inhibition of *NCED9* gene expression [102] (Supplementary Table 2), which is considered a key player in the control of seed germination and thermo-inhibition [103, 104]. It seems that the application of SL may restrict the inhibition of seeds germination in heat stress by limiting the ABA biosynthesis. Recently, the work of Chi and colleagues has shed new light on the relationship between the SL and ABA biosynthetic pathways in tomato plant responses to extreme temperature changes at the seedling phase. Exposure to 4 or 42 °C temperatures contributed to a significant upregulation of *CCD7*, *CCD8 and MAX1* genes in WT’s roots and leaves. Moreover, the number of transcripts detected was intrinsically higher in the roots than in leaf samples [105]. The pre-treatment of WT and *Slccd7* plants with GR24^5DS^ reduced sensitivity to heat stress, as evidenced by less serve wilting, lower relative electrolyte leakage values and malondialdehyde contents in the leaves of pre-treated plants compared to control plants. Further, SL-mediated extreme temperatures tolerance was revealed to be associated with the escalation of *NCED6* gene expression in tomato shoots, followed by increased ABA content in WT and *cdd7* tomato mutant. Moreover, the transcripts level was always lower in the mutant than in WT plants [105] (Supplementary Table 2). The opposite SL-ABA interactions were perceived with other SL biosynthesis mutants in monocotyledonous plants. Rice *d27* mutant seedlings display significantly decreased shoot ABA contents with lower transcripts amounts of ABA-responsive genes *MYB DOMAIN PROTEIN 2* (*MYB2*) and *RAB16C* and impaired cold tolerance abilities [80] (Supplementary Table 2). As the *D27* gene acts upstream of the *CCD7* gene in the SL biosynthesis pathway, the observed differences may result from the proposed role of the *D27* gene as a point connecting the SL and ABA biosynthetic pathways. This demonstrates that SL may modulate the ABA biosynthesis, influencing the ABA-dependent transcriptional responses during heat or cold stress conditions. Importantly, GR24^5DS^ treatment cannot rescue the severe wilting phenotype of ABA-deficient *notabillis* tomato plants under heat and cold stresses. What is more, the SL-induced activation of extreme temperatures resistance factors (*HEAT SHOCK PROTEIN 70 [HSP70], C-REPEAT BINDING FACTOR 1 [CBF1*]) was abolished in *notabillis* plants [83]. These indications prove that SL positively regulate tomato’s tolerance for heat and cold stresses in an ABA-mediated way. Hence, exogenous treatments or transgenic approaches for higher SL bioaccumulation may be potential strategies for developing tolerance to extreme temperatures in crops. However, it seems possible that the balance in ABA and SL levels may depend on the type of abiotic stress the plant is subjected to. For instance, Liu and coworkers showed that PEG-induced osmotic stress led to enhanced ABA accumulation in both shoot and roots of *Lotus japonicus*, while during the phosphate (Pi) starvation, ABA level remains untouched [106]. In contrast, SL biosynthesis is typically promoted while Pi deficiency occurs [107, 108]. Nonetheless, further research revealed that the simultaneous osmotic stress and Pi deficiency increased ABA accumulation in both *L. japonicus* organs. This could explain why increased amounts of SL under Pi deficiency even more intensify ABA biosynthesis [106]. An SL-deficient *Ljccd* RNAi line was subjected to soil Pi deficiency stress or in combination with osmotic stress to verify this hypothesis. Plants with a silenced expression of SL biosynthesis gene did not display remarkable differences in ABA concentrations in roots compared to WT genotype under Pi starvation. In contrast, surprisingly, an upregulation in ABA metabolism was noted in shoots and roots under combined stresses, compared to Pi starvation alone. Additionally, in the pre-treated roots with *rac*-GR24, ABA level persists low despite PEG (Supplementary Table 2). All the outcomes suggest that a limitation in SL production in the roots might be necessary to allow organ-dependent ABA production (Fig. 3). Actually, *LjNCED2* gene expression in WT escalated over time the PEG treatment, while the other genes from the NCED family were unaltered [106]. The discovery that *rac*-GR24 can inhibit upregulation of *LjNCED2* suggests that particular genes from the ABA biosynthesis pathway may be SL-sensitive during specific abiotic stresses. Similar observations were noted for two identified homologues *CCD8* homologues in tobacco (*NtCCD8A* and *NtCCD8B –* both biologically active) and their changes in the expression level after the ABA treatment or under the Pi starvation [109]. The Pi deficiency caused the increase in the transcripts level in both of the analyzed genes in root tissue, but the expression of *NtCCD8A* gene was six-fold higher than that of *NtCCD8B*. However, six hours after applying ABA, a three-fold increase in *NtCCD8B* transcripts level was detected, whereas *NtCCD8A* transcript levels were maintained. Obtained results suggest that different genes from the SL biosynthesis pathway may be regulated either by ABA levels or/and depend on the type of abiotic stress. Based on the relationships presented above, it appears reasonable to supplement the analyzes of SL/ABA accumulation in response to various abiotic stresses with an examination of the relative expression or mutations in the individual genes involved in hormone biosynthesis. However, also in this area of research, some inaccuracies may arise. The RT-qPCR analysis showed that rice *NCED1* gene expression in both drought tolerant and drought susceptible cultivars was progressively reduced with increasing water withholding stress, simultaneously with increasing ABA content [64]. In contrast, reports in other species like tomato [110] and barley [111] have evidenced that *NCED1* transcripts level is higher under drought stress than under control conditions. It would be interesting to detect if the function of individual genes in the *NCED* family may be species-dependent.

During the salt stress conditions, another player that may mediate the SL-ABA biosynthesis pathways interactions was revealed. Under control conditions, the expression of *CCD7* and *CCD8* homologues in arbuscular mycorrhizal (AM) *Sesbania cannabina* seedlings roots increased significantly after the ABA treatment and more interesting after the hydrogen peroxide (H_2_O_2_) application [112]. Similar observations were noted under salt stress conditions, where both SL-biosynthesis genes’ expression increased multi-fold. Furthermore, the germination assay of *P. ramosa* seeds induced by AM *S. cannabina* seedling root extracts revealed that under stress conditions, ABA-induced SL production was inhibited by a pre-treatment with dimethylthiourea (DMTU), which scavenges H_2_O_2_. On the contrary, ABA accumulation remains unaffected by DMTU. Hence, ABA appears to function upstream of H_2_O_2_ in ABA-induced SL accumulation in AM *S. cannabina* seedlings. Additionally, *rac*-GR24 contributed to rescuing the salt stress tolerance in the ABA-deficient plants. In contrast, ABA could only partially rescue the impaired salt stress tolerance in plants treated with tungstate (SL biosynthesis inhibitor) [112]. All this implies that ABA and SL work together to maintain salt stress tolerance in *S. cannabina* seedlings by ABA – H_2_O_2_ – SL pathway. Cooperation between SL and ABA biosynthesis pathways in salt stress was also noted in arabidopsis [60] and lettuce plants [113]. Most studies investigating the relationship between the ABA and SL biosynthetic pathways are related to drought stress however, current researches show many discrepancies. Water scarcity contributes to the activation of various defense mechanisms aimed at water retention in cells and organs. One of the best-known effects of plants against water loss is limiting transpiration by closing the stomata. This process is controlled by ABA, whose levels increase rapidly during drought stress. Moreover, the expression of SL biosynthesis genes in shoot also increased multi-fold times, followed by enhanced phytohormone accumulation in plants tissues, noted in several plant species, including arabidopsis [61] and tomato [93] (Fig. 3). In addition, plants harbouring mutations in the *CCD7* or *CCD8* genes display decreased drought tolerance due to ABA hyposensitivity at the guard cell level [61, 79, 84, 93]. However, the published results of experimental work aimed to determine the function of SL under water deficiency in arabidopsis were contradictory [83, 114]. While a slightly different experimental setup might explain some inconsistencies (different growth conditions, seedling age, and different periods of exposure to drought), it is puzzling that in one instance, SL biosynthesis mutants presented drought-sensitive phenotype [83], while in the other, their behaviour did not differ from the WT [114]. Ha and coworkers proved their results by hormone treatment of SL-depleted mutants and WT plants, rescuing the drought sensitive phenotype or enhancing the stress tolerance, respectively [83] (Supplementary Table 2). On the other hand, a study conducted on rice complements the presented issue and aligns with the results obtained by Bu and colleagues. Here, *CCD7* and *CCD8* rice mutants showed significantly higher survival rates than WT under drought stress [87]. Also, in support of this view, researchers detected enhanced ABA accumulation in the shoots of SL-depleted (*CCD7* and *CCD8*) rice plants, resulting in more efficient water retention because of accelerated closing of the stomata. In contrast rice *d27* mutant was unable to survive under the same drought conditions. Additionally the ABA levels in *d27* mutants were also lower than in the WT plants under drought [87]. The above-mentioned inconsistencies in the tolerance of SL-biosynthesis mutants to drought conditions may result from the different production of SL in monocotyledonous and dicotyledonous plants.

## Cross-talk between SL and ABA pathways at the perception level

Crosstalk between SL- and ABA-related genes is associated with the balance of endogenous hormones level, but also with changes in the sensitivity of plants to hormone presence. The fact that SL-depleted plants are hypersensitive to various environmental stresses and hyposensitive to ABA in the aspect of stomatal closure was corroborated in three genetically distant plant species, arabidopsis [61], tomato [93], and *L. japonicus* [84], by independent research groups. Therefore, it is also sufficient to elaborate on the relationship between SL and ABA signaling pathways. So far, little research has addressed the SL-ABA interplay at the signaling level under abiotic stress conditions. One of the presented issue’s first studies was carried out on arabidopsis F-box protein from the SCF complex – the *MAX2* gene [83, 114]. Two independent groups presented a novel function of the *MAX2* gene in plant drought response, expanding its role in an ABA-dependent manner. Arabidopsis *max2* mutant is hypersensitive to drought and evaporates more water than WT plants due to a thinner cuticle layer, increased stomatal density and inefficient stomatal closure caused by lower responsiveness to ABA [83, 114]. What is more, the qPCR analysis reveals that the relative transcripts level of ABA signaling, biosynthesis, transport, and catabolism genes were diminished in *max2* compared to WT seedlings under drought conditions [114] (Supplementary Table 3). In general, presented observations indicate that crosstalk between SL and ABA is prominent in the transduction of stress signals. However, the analyzes carried out on mutants in the genes encoding the F-box protein from the SCF complex (*AtMAX2*/*OsD3*) in terms of the functioning of the SL signaling pathway seem controversial due to the participation of these the F-box proteins in the signal transduction pathway of KAR [115], which engagement in drought stress tolerance was also elaborated [116].

Another experimental examined component from the SL-signaling complex in terms of ABA-related drought response is SL-repressor. It is expected that mutation in the SL-repressor should have the opposite effect on plant functioning to the SL-depleted or SL-insensitive plants due to the constantly active SL transduction pathway. In arabidopsis genome, three genes encoding SL repressors have been identified so far – *SMXL6*, *SMXL7* and *SMXL8* [117]. Characterizing single and double mutant combinations under drought stress revealed that knock-out of one of the SL-repressor genes makes no difference in the plant survival rate compared to WT, while mutations in two SMXL genes cause mild promotion of drought resistance [118]. The two different triple *smxl6/7/8* mutant lines exhibited significantly higher drought tolerance than WT (Supplementary Table 3). All these facts clearly highlight the functional redundancy of SMXL6,7,8 proteins acting as negative transcription regulators of SL signaling in arabidopsis. The increased drought tolerance of triple mutant was investigated in detailed physiological and biochemical analysis. Reduced cuticle permeability, increased anthocyanin biosynthesis, enhanced reactive oxygen species (ROS) detoxification capacity, and decreased water loss were detected, which might help *smxl6,7,8* mutant plants survive drought [118]. Additionally, the authors recorded higher expression levels of ABA INSENSITIVE 5 (ABI5) and SENESCENCE-ASSOCIATED GENE 29 (SAG29) genes after 2 and 4 h of dehydration in *smxl6,7,8* mutant than in WT plants. Both of these genes have been widely used as a marker gene for ABA response, thus suggesting that the increased tolerance of *smxl6,7,8* plants might be connected with ABA hypersensitivity. Notably, the increased sensitivity to ABA of the triple mutant compared to WT was also proved in both cotyledon opening and growth inhibition assay [118]. Analogous observations were noted in the case of arabidopsis plants harbouring a mutation in *SUPPRESSOR OF MAX2 1* (*SMAX1*) and *SMXL2* genes. SMAX1 and SMXL2 are components of the core signal transduction complex of the KAR, suppressing the activity of MAX2, which is a common point in both KAR and SL signaling pathways [119]. The *smax1/smxl2* mutant exhibited enhanced drought tolerance due to increased cuticle formation and ABA hypersensitivity, which was proved in assays of stomatal closure, cotyledon opening, chlorophyll degradation, and growth inhibition [120]. Since not all SL signaling transduction pathway components are SL-specific [115], it was postulated that mutants in the SL receptor D14 should be considered a gold standard in studies disclosing the role of SL in plants [121]. Barley *hvd14.d* mutant displayed hypersensitive to drought phenotype, illustrated by lower leaf relative water content (RWC), impaired photosynthesis, disorganization of chloroplast structure, altered stomatal closure and density [121] (Supplementary Table 3). The transcription profile of ABA signaling genes, including *HvPYL4*, *HvPP2C4*, *HvSnRK2.1* and *HvABI5* remain unchanged in *hvd14.d* mutant compared to WT under drought stress [121]. On the other hand, the expression of genes related to ABA biosynthesis, such as *HvNCED1*, *HvNCED2*, and *HvAo5b* was up-regulated in the mutants due to water deficit. The outcomes suggest that the mutant’s drought tolerance reduction is probably caused by an inability to respond to the elevated ABA levels and trigger a proper stress response [121]. Hence, it can be assumed that SL-insensitive plants show reduced ABA signal perception. Additionally, drought-sensitive phenotype and physiological deterioration caused by stress were also proved in the same research on arabidopsis *atd14-1* plants. The same plant drought hyposensitivity phenotype as in the case of *hvd14.d* and *atd14-1* was noted during independent research focused on *atd14-2*. In this study, loss-of-function of the *D14* gene was associated with lower anthocyanin content, delayed senescence, and slower ABA-mediated stomatal closure [122]. Overall, mutants in the SL biosynthetic and SL signaling genes have been shown to have a higher stomatal conductance than the WT in the presence or absence of abiotic stresses and an impaired response to ABA treatment [83, 87, 106, 114, 118, 121, 123]. Therefore, the participation of SL in proper guard cell functioning and adjusting plant responses to water deprivation is supported enough to consider SL as a crucial factor in determining the plants’ drought tolerance. Especially since the expression of *MAX2* and *D14* genes are wide and more enriched in the stomatal lineage than in other leaf tissue [124]. In addition, the simultaneous application of ABA and *rac*-GR24 resulted in a smaller diameter of stomata than that of ABA or *rac*-GR24 alone [124] (Supplementary Table 3).

Recently it was shown that treatment with GR24^5DS^ contributes to increasing plant’s drought tolerance by efficient stomata closure, followed by enhanced accumulation of *miR156* molecule in tomato leaves [125]. To date, several studies indicate the role of *miR156* and its targets belonging to the SQUAMOSA PROMOTER BINDING PROTEIN-LIKE (SPL) family in regulating stress tolerance [126–129]. To understand if the enhanced levels of *miR156* are a consequence of elevated SL shoot accumulation during drought, the SL-depleted plants were subjected to water deprivation. No induction of *miR156* biogenesis could be observed in *CCD7*-silenced plants under drought conditions compared to WT. Further analyses revealed that the overexpression of the *AtMIR156* gene led to higher ABA sensitivity [125]. In addition, the stomatal closure induced by ABA spraying was more pronounced in *miR156-oe* plants than in WT (Supplementary Table 3). Hence, researchers have shown that the *miR156* may be the connecting point of both ABA and SL signaling pathways in the aspect of stomata action [125] (Fig. 3). However, some studies indicate that SL may play an active role in the closure of the stomata in an ABA-independent way, which was proven in several plant species, including arabidopsis [124, 130], *Vicia faba* [131] and, grape [132]. Arabidopsis plants could close their stomata three hours after the *rac*-GR24 treatment in a dose-dependent manner [124]. In addition, the same observations were noted in the SL-induced closure of stomata in multiple various lines of ABA biosynthesis, receptors and signaling mutants. Because H_2_O_2_ is an essential secondary messenger in closing stomata, the participation of that molecule in SL-induced stomata responses was also investigated. Indeed, SL-induced stomata closure was utterly blocked in ascorbic acid or catalase presence, reducing the H_2_O_2_ amount in cells [124] (Supplementary Table 3). A similar effect was observed under the nitrogen oxide (NO) analysis, where the PTIO (an NO scavenger) and Na_2_WO_4_ (a nitrate reductase inhibitor) prevented SL-induced stomatal closure. Moreover, the analysis indicated that mutation in the *SLOW ANION CHANNEL-ASSOCIATED 1* (*SLAC1*) gene (a key player in ABA-induced stomatal closure) resulted in ABA and SL insensitivity, pinpointing that both hormone signaling pathways modulate the osmotic pressure by SLAC1, leading to the closure of stomata [126]. All together suggests that SL mechanisms leading to the closing of the stomata require the accumulation of both H_2_O_2_ and NO in the guard cells and activation of *SLAC1*, similar to ABA. Another study reveals that Ca^2+^ chelator and Ca^2+^ channel blockers strongly inhibit the SL-induced closure of stomata [130]. Through examining a collection of calcium-dependent protein kinase (CPK) mutants, the CPK33 protein was identified as a potential Ca^2+^ transducer involved in SL-mediated stomata response. The *cpk33* mutant was impaired in SL-, H_2_O_2_- and Ca^2+^-induced stomatal closure. Thus researchers propose that SL stimulate the production of H_2_O_2_ that possibly activates the Ca^2+^ transducer CPK33 which likely modulates anion and potassium channels to promote stomatal closure. In contrast to all the presented data above, there is one study where treatments with a SL analogue cannot induce stomatal closure in arabidopsis [133] however, conductivity analysis was performed within one hour after SL treatment, which may not be sufficient time to observe a physiological effect.

## Organ-specific dynamics of SL and ABA relations

**Fig. 4:**
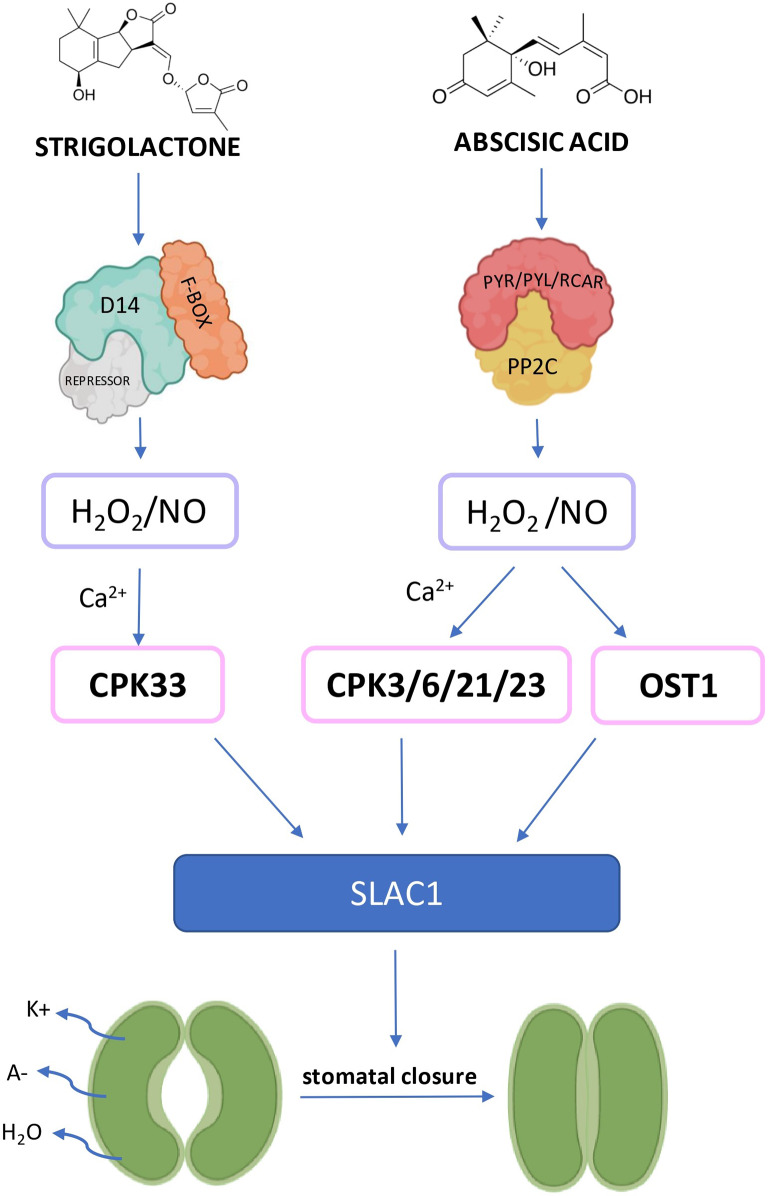
SLAC1 is a common point providing stomatal closure in SL- and ABA-dependent ways. Both SL and ABA signaling pathways initiate the production of secondary messengers for stomata movement, namely H_2_O_2_ and NO. These molecules indirectly activate calcium-dependent (CPK) or calcium-independent kinases (OST1), which provide the phosphorylation signal promoting conformational changes of SLAC1 and outflow of anions (A-) outside the guard cell. Further, with the outflow of cations (K+) from the cell, the ionic strength outside the guard cell increases, followed by H_2_O outflow. The turgor of the guard cell decrease, which leads to stomatal closure. PYR - PYRABACITN RESISTANCE, PYL - PYRABACTIN RESISTANCE 1-LIKE, RCAR - REGULATORY COMPONENT OF ABA RECEPTOR, PP2C - PROTEIN PHOSPHATASE 2 C, CPK – CALCIUM-DEPENDENT KINASE, D14 – DWARF 14, OST1 – OPEN STOMATA 1, SLAC1 – SLOW ANION CHANNEL-ASSOCIATED 1. Created with BioRender.com

The studies above clearly indicate the interaction between the ABA and SL biosynthesis and signaling pathways under control conditions and response to various abiotic stresses, especially drought or salinity. In particular, previous experimental research on arabidopsis, tomato and, *L. japonicus* allowed proposing a model connecting SL and ABA levels in a root-shoot-dependent manner during drought stress [125, 134]. In this model, the drop in SL biosynthesis in the roots may be required to empower ABA production. In this context, SL might play a sensor role in water deprivation, then promote the ABA accumulation in root tissue. Indeed, under water scarcity, ABA accumulation in root tissues, followed by increased ABA content in the shoot, is closely correlated with a decrease in leaf stomatal conductance [135] or alleviates stress by other mechanisms [136] (Fig. 3). Referring to the presented model, it is believed that inhibited shootward flow of SL may trigger SL biosynthesis in shoots by an unknown mechanism. Especially since greater amounts of SL are produced in the roots, hormone molecules are probably more intensively transported to the shoot under optimal conditions. Under stress, the enhanced regulation of SL biosynthesis genes in the above-ground organs of various plant species may suggest that SL play an active role in overcoming harsh environmental conditions and increasing plants’ survival rate. The enhanced activation of SL biosynthesis genes in shoots was proved by transcript quantification during stress in several plant species, such as arabidopsis [61], tomato [93] and rice [87]. What is more, using a reciprocal grafting approach between SL-deficient mutants and WT plants, it was demonstrated that stomatal closure is affected by the shoot genotype rather than the root genotype. WT tomato scions grafted onto SL-depleted rootstock exhibited an increased amount of SL biosynthetic genes’ transcripts, as well as lower transpiration phenotype under drought compared to control grafted plants [123]. Further analysis revealed that the more efficient closure of the stomata was due to enhanced sensitivity to endogenous ABA, rather than an increase in total free ABA. Similarly, previous data related to *L. japonicus* indicate no changes in ABA accumulation in shoots of SL-depleted plants under osmotic stress compared to WT [106], which suggests that SL-ABA relations in above-ground organs might occur at the perception level. However, tomato and *L .japonicus* studies were conducted on plants harbouring the mutation in *CCD7* gene. In contrast, one research that proves that under drought stress, the mutation in *CCD7* and *CCD8* genes led to increased ABA accumulation in leaves, in opposition to *d27* mutation, where the ABA content decreased significantly compared to control plants [87]. Unfortunately, the research was carried out on rice, the monocot specie. To date, no evidence confirms a similar relationship in dicots plants during drought conditions. Therefore, the *D27* gene should be included in analysing the SL-ABA crosstalk in dicots under stress. The unchanged ABA levels compared to WT plants were also noted in barley SL-insensitive *hvd14.d* mutant under dehydration conditions [121]. A few additional players contributing to the closure of the stomata, including H_2_O_2_, NO, miRNA156, *SLAC1* and *CPK33* in either ABA-dependent or ABA-independent ways, were identified. It was proposed that SL may trigger the ABA sensitivity in guard cells by the interaction between miR156 and SL repressor protein [137]. Under optimal environmental conditions, the presence of SMXL6,7,8 transcriptional repressors inhibits the *miR156* biogenesis. In turn, the SPL transcription factors may accumulate, maintaining the ABA sensitivity at the low level and opening stomata. In contrast, under drought conditions, the activation of SL biogenesis, followed by assembling the SL signaling complex, leads to the degradation of SMXL6,7,8 proteins. Consequently, the *miR156* molecules may accumulate and inhibit mRNA translation, thus blocking the formation of SPL native proteins. This molecular cascade is believed to increase the sensitivity of guard cells to ABA and accelerates their closure (Fig. 3). On the other hand, combining previous research of SL-induced closure of stomata in ABA-independent way the mechanism might be based on the activation of SLAC1 by H_2_O_2_/NO and CPK33 pathway. It was proved that SL biosynthesis and further SL signaling lead to H_2_O_2_ and NO production. Next, activation of SLAC1 modulates the osmotic pressure in guard cells, leading to the closure of stomata [124]. In addition, another study revealed that CPK33 is required for SL-modulated proper stomata functioning [130]. It is important that the *cpk33* mutant is impaired in H_2_O_2_-induced stomatal closure, but not in SL-mediated H_2_O_2_ production. This clearly highlights that CPK33 acts downstream upon H_2_O_2_/NO in SL-induced stomata regulation. It was also shown that in arabidopsis guard cells, anion channel SLAC1 is regulated by CPK proteins [138]. Thus, the SL-induced regulation of closing the stomata under drought might be activated by SL – H_2_O_2_/NO – CPK33 – SLAC1 pathway (Fig. 4). It is puzzling that CPK33 was reported as a negative regulator of slow anion channels activity in ABA-induced stomatal closure [139, 140], unlike where the *CKP33* gene with mutation blocked SL-induced stomata regulation, clearly indicating the role of CPK33 as a positive SL-mediated stomatal regulator. During ABA-dependent pathway, the SLAC1 might be activated either by calcium-independent kinases, such as OPEN STOMATA 1 (OST1) or CPK proteins [141] (Fig. 4). Under water-deficit, stress can trigger ROS accumulation and promote activation of Ca^2+^ channels, resulting in increased Ca^2+^ in the cytoplasm of guard cells [142]. CPK then perceives the Ca^2+^ cations to validate signal transduction. The phosphorylation signal promotes the conformation changes of SLAC1, thus enabling the outflow of anions outside the guard cell. Further, with the outflow of cations from the cell, the ionic strength outside the guard cell increases, followed by H_2_O outflow. The turgor of the guard cell decrease, which leads to stomatal closure. The role of a positive calcium-dependent kinase regulator of ABA-mediated stomata closure was experimentally proved for several CPK proteins, including CPK3/6/21/23 (Fig. 4) [143]. However, mutation of *CPK33* resulted in arabidopsis the ABA-dependent hyperactivation of SLAC1, while the *CPK33* overexpression showed opposite phenotype [139, 140]. Taken together, the CPK33 might be an essential player in both ABA- and SL-dependent control of stomata closure. Nevertheless, the discrepant role of CPK33 in guard cell ABA and SL signaling is needed to be further unraveled. Presented results indicate that SL and ABA crosstalk dynamics at the biosynthesis and perception level are seemingly opposite in the above- and below-ground organs, reinforcing the need to separate roots and shoots analysis when addressing issues related to SL-ABA interactions under stress.

## Main open questions and future goals

The primary hormone associated with the plant response to drought stress is ABA [144]. With an increase in experimental data indicating the participation of SL in maintaining stress tolerance, it is expected that SL might interplay, directly or indirectly, with ABA in regulating adaptive stress responses in plants. Thus, the crosstalk between SL and ABA’s biosynthetic and signaling pathways during abiotic stresses is eagerly investigated. At the biosynthesis level, the SL-ABA relations in roots are pretty well documented regarding growth and developmental processes or in response to abiotic stresses. However, some inconsistencies exist in the metabolic SL-ABA interplay at the shoot level. There is an open question if SL may trigger ABA biosynthesis in response to drought or whether the SL-ABA crosstalk is related only to perception level. Beyond the above observations, which suggest that the influence of SL and ABA on their mutual concentrations may be more or less intimate in different species and organs, more and more research is focusing on the crosstalk between the signaling pathways of both hormones. First, the mechanism underlying root-to-shoot communication at the SL level requires in-depth investigation. It is tempting to see how the decreased levels in roots might contribute to the activation of SL biosynthesis in leaves. Finally, it would be interesting to experimentally confirm the relations between SL-repressor and *miR156* leading to enhanced ABA sensitivity, as was recently proposed [137].

## Electronic supplementary material

Below is the link to the electronic supplementary material.

Supplementary Material 1: Supplementary Table 1: SL-ABA under control conditions. Table summarizing interactions of SL-ABA biosynthesis under control conditions.

Supplementary Material 2: Supplementary Table 2: SL-ABA biosynthesis under stress conditions. Table summarizing interactions of SL-ABA biosynthesis under stress conditions.

Supplementary Material 3: Supplementary Table 3: SL-ABA percepcion under stress conditions. Table summarizing interactions of SL-ABA signaling under stress conditions.

## Data Availability

All data generated or analysed during this study are included in this published article [and its supplementary information files].

## List of abbreviations

AAO: Abscisic acid oxidase
ABA: Abscisic acid
ABA-GE: ABA-glucosyl ester
ABI: Abscisic acid insensitive
ABRE: ABA-responsive element
AM: Arbuscular mycorrhizal
AUX: Auxins
BR: Brassinosteroids
BRC: Branched
CBF: C-repeat binding factor
CCD: Carotenoid cleavage dioxygenase
CKs: Cytokinins
CLA: Carlactonoic acid
CLAMT: Carlactonoic acid methyltransferase
CPK: Calcium-dependent protein kinase
D: Dwarf
DAD: Decreased apical dominance
DMTU: Dimethylthiourea
ET: Ethylene
GA: Gibberellins
HSP: Heat shock protein
HTD: High-tillering dwarf
JA: Jasmonates
KAI: Karrakin insensitive
KAR: Karrikins
MAX: More axillary growth
MeCLA: Methyl carlactonoate
MYB: Myb domain protein
NCED: Epoxycarotenoid dioxygenase
NO: Nitrogen oxide
NSY: Neoxantin synthetase
OST: Open stomata
Pi: Phosphate
PAP: Production of anthyocyanin pigment
PEG: Polyethylene glycol
PP2Cs: Protein phosphatase 2 C
PYL: Pyrabactin resistance 1-Like
PYR: Pyrabactin resistance
RCAR: Regulatory component of aba receptor
RMS: Ramousus
RWC: Relative water content
SCF: SKP1-Cullin-F-Box
SLAC: Slow anion channel-associated
SL: Strigolactones
SMAX: Suppressor of Max2
SMXL: Supressor of Max2 1-Like
SnRK2s: Sucrose nonfermenting 1 related protein kinases 2
SPL: Squamosa promoter binding protein-like
T: Tillering
TCP: TCP domain protein
TF: Transcription factor
TI_50_: 50% inhibition of seed germination by thermo-inhibition
UPS: Ubiquitin proteasome system
WT: Wild type
XD: Xanthoxin dehydrogenase
ZEP: Zeaxanthin epoxidase
