# Supplementary material for: Strigolactones and abscisic acid interactions affect plant development and response to abiotic stresses
Source: BMC Plant Biol. 2023 Jun 13;23:314. doi: 10.1186/s12870-023-04332-6 (PMC10262459; doi:10.1186/s12870-023-04332-6)
Supplement: Supplementary file 1 — Supplementary Material 1: Supplementary Table 1: SL-ABA under control conditions. Table summarizing interactions of SL-ABA biosynthesis under control conditions. [file 12870_2023_4332_MOESM1_ESM.docx]

| specie | | genotype | age of plant | treatment | effect | ref. |
| --- | --- | --- | --- | --- | --- | --- |
| *Arabidopsis thaliana* | WT Collumbia-0 | | 2-week-old seedlings | 100 µM of ABA | 4 and 8-fold higher expression of *CCD7*and *CCD8* genes in leaves, respectively 10h after treatment | 83 |
|  | *pAtD27:NLS-GUS* | | 7-day-old seedings | 50 μM of ABA for 6 h | 1.5-fold higher expression of *D27* gene, observed GUS activity in primary and lateral roots | 82 |
|  | *d27* | | 2-week-old seedlings | - | 20% reduced ABA shoot content compared to WT, no significant changes detected in root samples |  |
|  | *35S::GmMAX1a* | | 2-week-old seedlings | - | ~45% increased shoot ABA content | 92 |
|  | *35S::GmMAX3b* | |  |  | ~450% increased shoot ABA content |  |
|  | *35S::GmMAX4a* | |  |  | ~520% increased shoot ABA content |  |
|  | *atmax1* | |  |  | ~45% reduced shoot ABA content |  |
|  | *atmax3* | |  |  | ~35% reduced shoot ABA content |  |
|  | *atmax4* | |  |  | ~65% reduced shoot ABA content |  |
|  | *pAtD27::HvD27* in *atd27* background | | 6-week-old seedling | - | ~65% increased leaf ABA content compared to WT | 91 |
| *Hordeum vulgare* | RNAi-mediated down-regulation of two ABA catabolic genes (HvABA8’OH-1 and HvABA8’OH-3) in Golden Promise background, 2 independent lines: LOHi236 LOHi272 | | 14-week-old plants | - | LOHi236: ~0.75-, 0.42, 0.64, 0.37-fold lower expression of *HvD27*, *HvCCD7*, *HvCCD8* and *HvMAX1* genes in root tissue, respectively  LOHi272: ~0,64, 0.5, 0.62-fold lower expression of *HvCCD7, HvCCD8* and *HvMAX1* genes in root tissue, respectively |  |
| *Oryza sativa* | WT Indica, 93-11 line | | 2-week-old seedlings | 5 μM of rac-GR24 for 6 and 12 h | Shoot: ~2.5-fold increased expression of *NCED1* after 6h*, ~*4-fold increased expression of *NCED2* after 12h Roots: ~2.2- and ~1.8-fold increased expression of *NCED3* after 6 and 12h | 80 |
|  |  |  |  | 50 μM of ABA for 3, 6 and 12 h | *OsCCD7*: ~0.45-fold lower expression 12h after treatment;  *OsCCD8: ~*0.7, 0.8 and 0.9-fold lower expression 3h, 6h, 12h after treatment; *OsD27*: *~*0.45, 0.8 and 0.8-fold lower expression 3h, 6h, 12h after treatment; *OsD53*: ~0.55, 0.6-fold lower expression 6h and 12h after treatment |  |
|  | *t20* | | 2-week-old seedlings | - | 40% reduction of SLs content in roots,  33% reduction of ABA content in roots,  24% reduction of ABA content in shoots |  |
|  | *OsD27* overexpression lines  (*D27-OE1* and *D27-OE2*) | | 24-day-old seedlings | - | 96% and 32% increased shoot ABA content in *D27-OE1* and *D27-OE2* lines, respectively | 87 |
|  | *d27* | | 5-leaf stage plants |  | shoot ABA levels compared as in WT plants, |  |
|  | *ccd7* | |  |  | 150% increase of shoot ABA content ~1.35-fold increased expression of *D27* gene |  |
|  | *ccd8* | |  |  | 150% increase of shoot ABA content ~1.33-fold increased expression of *D27 gene* |  |
| *Phelipanche ramosa* | WT | | seeds, germination phase | 10^-9^ M GR24 treatment for 3 days | ~28-, ~50 and ~100-fold increase of *PrABA8’OH-1* gene expression 6h, 12h and 18h after treatment; 87,5% decrease in ABA content | 93 |
| *Solanum lycopersicum* | WT Money Maker | | 4-week-old seedlings | 50 μM of the ABA biosynthesis inhibitor abamineSG for 7 days | 58% reduction of SLs content in root exudates; 23% lower root ABA content | 84 |
|  |  |  |  | 50 μM of the SL biosynthesis inhibitor D2 for 7 days | 58% reduction of SLs content in root exudates |  |
|  | *notabilis* | |  | - | 45% lower root ABA content,  40% reduction of SLs content in root exudates |  |
|  | *sitiens* | |  |  | 60% lower root ABA content,  47% reduction of SLs content in root exudates |  |
|  | *flacca* | |  |  | 65% lower root ABA content,  52% reduction of SLs content in root exudates |  |
| *Vitis vinifera* | WT Barbera | | mature plant with grape bunches at the early stage of ripening | sprying with 10 μM of the rac-GR24, or 200 μM of ABA | ~15% lower content of soluble solids (glucose and fructose) 144h after the treatment  ~35% lower anthocyanins content 144h after treatment (compared to ABA treated WT) | 95 |
| *Zea mays* | *nced1* | | 8/11-day-old seedlings | 0.2 mM of ABA | ~90% reduced seed germination of *Striga hermonthica* | 85 |

Supplementary table 1. SL-ABA interactions under control conditions
