# Supplementary material for: Strigolactones and abscisic acid interactions affect plant development and response to abiotic stresses
Source: BMC Plant Biol. 2023 Jun 13;23:314. doi: 10.1186/s12870-023-04332-6 (PMC10262459; doi:10.1186/s12870-023-04332-6)
Supplement: Supplementary file 2 — Supplementary Material 2: Supplementary Table 2: SL-ABA biosynthesis under stress conditions. Table summarizing interactions of SL-ABA biosynthesis under stress conditions. [file 12870_2023_4332_MOESM2_ESM.docx]

| specie | genotype | age of plant | applied stress | treatment | effect | ref. |
| --- | --- | --- | --- | --- | --- | --- |
| *Arabidopsis thaliana* | WT Columbia-0 | seeds, germination phase | heat stress | 20 μM of GR24 | TI_50_=29,1°C TI_50_+GR24=32,5°C,  ~0,5-fold lower expression of *NCED9* gene compared to non- treated seeds | 102 |
|  | *ccd7* |  |  |  | TI_50_=26,8°C TI_50_+GR24=31,9°C |  |
|  | *max1* |  |  |  | TI_50_=26,2°C TI_50_+GR24=32,4°C |  |
|  | *max2* |  |  |  | TI_50_=24,3°C TI_50_+GR24=24,9°C |  |
|  | WT Columbia-0 | 24-day-old seedlings | dehydration stress for 6h | - | LEAVES: ~12 and 8-fold higher expression of *CCD7* and *CCD8* gene, respectively | 83 |
|  | *ccd7* | 3-week-old seedlings | - | 30 μM of ABA | ~45% wider stomatal aperture 1h after treatment (compared to ABA-treated WT) |  |
|  |  |  | drought (13 days) + 3 days of rehydration | - | ~0,1-fold lower survival rate |  |
|  |  |  |  | 5 μM of rac-GR24 | ~90% survival rate |  |
|  | *ccd8* |  | - | 30 μM of ABA | ~65% wider stomatal aperture 1h after treatment (compared to ABA-treated WT) |  |
|  |  |  | drought (13 days) + 3 days of rehydration | - | ~0,1-fold lower survival rate |  |
|  |  |  |  | 5 μM of rac-GR24 | ~100% survival rate |  |
|  | WT Columbia-0 | 2-week-old seedlings | water withholding for 6h | - | ~50% water loss for all tested genotype | 115 |
|  | *ccd7* |  |  |  |  |  |
|  | *ccd8* |  |  |  |  |  |
| *Hordeum vulgare* | WT  Misato Golden | 3-week-old seedlings | water withholding for 3 days | - | ~3-fold higher expression of *NCED1* gene | 111 |
| *Lotus japonicus* | WT  Gifu B-129 | 4-week-old seedlings | osmotic stress (10 % PEG) for 6 days | - | ROOT: ~50% increase of ABA content; SHOOT: ~315% increase of ABA content | 106 |
|  |  |  | P starvation for 6 days |  | ROOT: no changes in ABA content; SHOOT: no changes in ABA content |  |
|  |  |  | P starvation + osmotic stress  (10 % PEG) for 6 days |  | ROOT: ~180% increase of ABA content; SHOOT: ~440% increase of ABA content |  |
|  |  |  | osmotic stress (10 % PEG) for 1 and 2 days | Pre-treatment with 5 µM of GR24 | ROOTS: 1d - 40% reduction of ABA content, ~0,25-fold lower expression of *NCED2* gene; 2d – 45% reduction of ABA content, ~0,8-fold lower expression of *NCED2* gene (compared to non-treated plants in time-course) |  |
|  | *ccd7* |  | P starvation for 6 days | - | ROOT AND SHOOT: no changes in ABA content  (compared to WT in stress conditions) |  |
|  |  |  | P starvation + osmotic stress (10 % PEG) for 6 days |  | ROOT: ~260% increase of ABA content; SHOOT: ~300% increase of ABA content (compared to *cdd7* in P starvation) |  |
|  |  |  | - | 5 µM of ABA | ~2-fold slower response to ABA compared to WT |  |
| *Nicotiana tabacum* | WT  Honghuadajinyuan | 4-week-old seedlings | P starvation for one week | - | ~350-fold increase of expression of *CCD8A gene,* ~60-fold increase of expression of *CCD8B* gene, (compared to control conditions) | 109 |
|  |  |  | - | 100 µM of ABA for 6h | ~expression of *CCD8A* gene untouched, ~3-fold increase of expression of *CCD8B* gene, (compared to plants treated with control solution) |  |
| *Oryza sativa* | WT, drought tolerant N22 | anthesis stage  (65 days after transplanting) | water withholding for 4 days | - | LEAF: ~100% reduced expression of *NCED1* gene, ~420% increased ABA content ROOT: ~0,3-fold lower expression of *NCED1* gene, ~250% increased ABA content | 86 |
|  | WT, drought susceptible IR64 |  |  |  | LEAF: ~100% reduced expression of *NCED1* gene, ~350% increased ABA content ROOT: ~0,2-fold lower expression of *NCED1* gene, ~250% increased ABA content |  |
|  | *d27* | 2-week-old seedling | - | - | ~15% reduced shoot ABA content compared to WT;  ~0,8 and 0,85-fold lower expression of *MYB2* and *RAB16C* genes, respectively | 80 |
|  | *ccd8* | 5-leaf stage | drought (10/12* days) | - | ~20% increased shoot ABA content, ~4,5-fold higher survival rate* (compared to WT in drought condition) | 87 |
|  | *ccd7* |  |  |  | ~40% increased shoot ABA content), ~4,25-fold higher survival rate* (compared to WT in drought condition |  |
|  | *d27* |  |  |  | ~40% reduced shoot ABA content, ~0% survival rate* (compared to WT in drought condition) |  |
| *Sesbania cannabina* | WT | 3-weeks-old seedlings | - | 10 mM of H_2_O_2_ for 10 days | ~11,5 and 3,8-fold increased expression of *CCD7* and *CDD8* genes in root, respectively | 112 |
|  |  |  |  | 100 μM of ABA for 10 days | ~11,5 and 3,5-fold increased expression of *CCD7* and *CDD8* genes in root, respectively |  |
|  |  |  | salt stress (200 mM of NaCl for 10 days) | - | ~10 and 3,5-fold increased expression of *CCD7* and *CDD8* genes in root, respectively |  |
|  |  |  |  | 5 mM of DMTU as a pre-treatment applied 24h before salt stress | ~60% reduction of germination of *P. ramosa* seeds; no changes in ABA content in whole seedling |  |
| *Solanum lycopersicum* | WT Castlemar and Aisla Craig | 4-month-old plants | water withholding for 24h | - | ~35-fold and ~25-fold increased expression of *NCED1* *gene* in roots of Castlemar and Aisla Craig, respectively | 110 |
|  | WT  Aisla Craig | 5-week-old seedlings | 4/42°C for 6 hours | - | 1. ROOT:, *CDD7:* ~12/8-fold increase*; CDD8:* ~-/8,5-fold increase*;  MAX1:* ~2,5/3,5-fold increase;  2. LEAF: *CDD7:* ~3,5/3,5-fold increase*; CDD8:* ~4/6,5-fold increase*;  MAX1:* ~4/3-fold increase; (compared to WT in control conditions) | 105 |
|  |  |  | 4/42°C for 12 hours |  | LEAF: ~90/150% increased ABA content, *NCED6*: ~3/2-fold increased, (compared to WT in control conditions) |  |
|  |  |  |  | pre-treatment with 3 μM of GR24^5DS^ | LEAF: ~90/415% increased ABA content, ~4,5/3-fold increased expression of NCED6 (compared to WT in control conditions) |  |
|  | *ccd7* |  |  | - | LEAF: ~165/165% increased ABA content, ~2/1,5-fold increased expression of NCED6, (compared to WT in control conditions) |  |
|  |  |  |  | pre-treatment with 3 μM of GR24^5DS^ | LEAF: ~170/260% increased ABA content, ~4/2,5-fold increased expression of NCED6, (compared to WT in control conditions) |  |
|  | *notabillis* |  |  | pre-treatment with 3 μM of GR24^5DS^ | HEAT: ~0.55-fold change in expression of *HSP90* gene COLD: 0.44-fold change in expression of *CBF1* gene |  |
|  | WT/WT  (shoot/root) M82 | 6-week-old seedlings | drought (3-5 days) | - | SHOOT: ~10- and 10-fold higher expression of *CCD7* gene 3 and 5 days under drought, respectively; ~10- and 3-fold higher expression of *CCD8* gene 3 and 5 days under drought, respectively; (compared to WT/WT in control conditions) | 114 |
|  | WT/*cdd7*  (shoot/root) |  |  |  | SHOOT: ~2,6 and 1,75-fold higher expression of *CCD7* 3 and 5 days under drought, respectively; ~0,65 and 1,7-fold changed expression of *CCD8* gene 3 and 5 days under drought, respectively; (compared to WT/WT in drought conditions) |  |
|  |  |  |  |  | ~30% lower stomatal conductance |  |
|  |  |  |  | 5 μM of ABA | ~40% faster response to ABA |  |
|  | *ccd7/ccd7*  (shoot/root) |  |  |  | ~2-fold slower response to ABA |  |

Supplementary table 2. SL-ABA biosynthesis interactions under stress conditions
