# Supplementary material for: Strigolactones and abscisic acid interactions affect plant development and response to abiotic stresses
Source: BMC Plant Biol. 2023 Jun 13;23:314. doi: 10.1186/s12870-023-04332-6 (PMC10262459; doi:10.1186/s12870-023-04332-6)
Supplement: Supplementary file 3 — Supplementary Material 3: Supplementary Table 3: SL-ABA percepcion under stress conditions. Table summarizing interactions of SL-ABA signaling under stress conditions. [file 12870_2023_4332_MOESM3_ESM.docx]

| specie | genotype | age of plant | applied stress | treatment | effect | ref. |
| --- | --- | --- | --- | --- | --- | --- |
| *Arabidopsis thaliana* | WT Columbia-0 | 4/6-week-old plants | - | 3h incubation with 0.1 or 1 µM of GR24 | ~25% and 40% reduced stomatal aperture | 124 |
|  |  |  |  | 3h incubation with 10 µM of ABA | ~45% reduced stomatal aperture |  |
|  |  |  |  | 3h incubation with 1 µM of GR24 and 10 µM of ABA | ~60% reduced stomatal aperture |  |
|  |  |  |  | 3h incubation with 1 µM of GR24 + pretreatment with 100 μM of ASA or 100 units/ml of CAT | stomatal aperture compared to non-treated WT |  |
|  |  |  |  | 3h incubation with 1 µM of GR24 + pretreatment with 200 μM of c-PTIO or 100 μM of Na_2_WO_4_ |  |  |
|  | *aba1, aba2, aba3, nced3nced5, pyr1pyl1pyl2pyl4, abi1, abi2, mpk3, mpk6, mpk9, mpk12, ost1* |  |  | 3h incubation with 1 µM of GR24 | ~35-45% reduced stomata aperture |  |
|  | *slac1* |  |  |  | stomatal aperture compared to non-treated WT |  |
|  | *max2* | 3-week-old seedlings | drought (13 days) + 3 days of rehydration | - | ~0,2-fold decrease survival rate | 83 |
|  | *max2* | 2-week-old seedlings | drought (1h) | - | reduced expression of ABA-related genes: ~0.3 - 0,6-fold decrease of *NCED3, CYP707A3,*  *ABCG22, HAB1, ABI1, ABI2* | 115 |
|  | *smxl6* | 3-week-old plants | drought (15 days) + 5 days of rehydration | - | comparable survival rate as WT (50%) | 119 |
|  | *smxl7* |  |  |  | comparable survival rate as WT (50%) |  |
|  | *smxl8* |  |  |  | comparable survival rate as WT (60%) |  |
|  | *smxl6,7* |  |  |  | 2-fold higher survival rate |  |
|  | *smxl6,8* |  |  |  | 1,4-fold higher survival rate |  |
|  | *smxl7,8* |  |  |  | 1,4-fold higher survival rate |  |
|  | *smxl6,7,8* |  |  |  | 6,2 – 8,2-fold higher survival rate, 212% higher RWC |  |
|  |  |  | dehydration (2h) |  | ~1,9-fold higher expression of *ABI5* gene |  |
|  | *d14* | 3-week-old seedlings | drought (15 days) | - | ~0,55-fold lower survival rate (compared to WT in drought conditions), | 123 |
|  |  |  | - | 30 µM of ABA | ~15% bigger stomatal aperture |  |
|  |  | 10-day-old seedlings | water withholding for 5 days | - | ~50% reduction of anthocyanin content |  |
|  | WT  Columbia-0 | 4/6-week-old plants | - | 3h incubation with 1 µM of GR24 + 2 mM EGTA / 1 mM LaCl_3_ / 1 µM AlCl_3_ | 25% bigger stomatal aperture for all EGTA, LaCl_3,_ AlCl_3_ treatments (compared to GR24-treated WT) | 130 |
|  | *cpk33* |  |  | 3h incubation with 1 µM of GR24 | ~37% bigger stomatal aperture (compared to treated WT) |  |
|  |  |  |  | 3h incubation with 1 mM of Ca^2+^ | stomatal aperture at the same level as non-treated WT |  |
|  |  |  |  | 3h incubation with 500 mM of H_2_O_2_ | ~40% bigger stomatal aperture (compared to treated WT) |  |
|  | WT  Columbia-0 | 4/6-week-old plants | - | 5 µM of GR24 | after 1h stomatal aperture at the same level as non-treated WT | 133 |
| *Hordeum vulgare* | *d14* | 2-week-old seedlings | drought (10 days) | - | ~90% reduction of RWC, ~16% and ~22% higher stomatal density in adaxial and abaxial side, respectively, ~65% reduced thickness of cuticle, ~4.5, 3, 2.5-fold higher expression of *NCED1*, *NCED2* and *Ao5b* genes, no changes in shoot ABA content, (compared to WT in drought condition), | 122 |
| *Solanum lycopersicum* | WT M82 | 4-week-old plants | - | 5 µM of GR24^5DS^ | ~40% and 30% reduced stomatal conductance 2h and 24h after treatment, respectively; 1.25, 3.5, 5.8-fold higher amount of mature miR156 2, 6 and 24h after the treatment | 125 |
|  |  |  | drought (15 days) | - | ~11,5-fold higher amount of mature miR156 |  |
|  | *cdd7* |  |  |  | ~0.3-fold lower amount of mature miR156  (compared to WT under drought) |  |
|  | *miR156-ox* |  | - | 1, 5 and 10 µM of ABA | ~30%, 45%, 50% reduced stomatal conductance 1h after the treatment (compared to treated WT) |  |

Supplementary table 3. SL-ABA signaling interactions under stress conditions
